# Novel Concept of CD4-Mediated Activation of Regulatory T Cells for the Treatment of Graft-Versus-Host Disease

**DOI:** 10.3389/fimmu.2017.01495

**Published:** 2017-11-08

**Authors:** Janine Schlöder, Carsten Berges, Andrea Tuettenberg, Helmut Jonuleit

**Affiliations:** ^1^Department of Dermatology, University Medical Center of the Johannes Gutenberg-University, Mainz, Germany

**Keywords:** regulatory T cell, graft-versus-host disease, cellular therapy, tolerance, CD4 stimulation

## Abstract

Allogeneic hematopoietic stem cell transplantation is the only curative treatment option for several hematological malignancies and immune deficiency syndromes. Nevertheless, the development of a graft-versus-host disease (GvHD) after transplantation is a high risk and a severe complication with high morbidity and mortality causing therapeutic challenges. Current pharmacological therapies of GvHD lead to generalized immunosuppression followed by severe adverse side effects including infections and relapse of leukemia. Several novel cell-based immunomodulatory strategies for treatment or prevention of GvHD have been developed. Herein, thymus-derived regulatory T cells (tTreg), essential for the maintenance of peripheral immunologic tolerance, are in the focus of investigation. However, due to the limited number of tTreg in the peripheral blood, a complex, time- and cost-intensive *in vitro* expansion protocol is necessary for the production of an efficient cellular therapeutic. We demonstrated that activation of tTreg using the CD4-binding human immunodeficiency virus-1 protein gp120 leads to a substantially increased suppressor activity of tTreg without the need for additional expansion. Gp120-activated tTreg prevent GvHD development in a preclinical humanized mouse model. In addition, gp120 is not only effective in prevention but also in therapy of GvHD by suppressing all clinical symptoms and improving survival of treated mice. These data indicate that tTreg activation by gp120 is a feasible and potent strategy for significant functional improvement of tTreg as cellular therapeutic for GvHD treatment without the need of complicated, time-intensive, and expensive *in vitro* expansion of isolated tTreg.

## Introduction

Allogeneic hematopoietic stem cell transplantation (aHSCT) is a potentially curative therapy for many patients with hematological malignancies or immune deficiencies. However, the development of graft-versus-host disease (GvHD) dramatically limits the efficacy of aHSCT, and is the leading cause of long-term morbidity and mortality (1). The report of the Worldwide Network for Blood and Marrow Transplantation in 2015 revealed an exponential increase in the use of HSCT, from the first transplant in 1957 to more than one million worldwide by now, with the highest number of HSCT reported in Europe [501,315 (52%), of which 45% were aHSCT] (2). Current therapeutic treatments for GvHD after aHSCT are primarily based on broadly immune suppressive agents such as corticosteroids and calcineurin inhibitors (3). However, GvHD still occurs in 40–60% of recipients with 50% of the patients developing steroid resistance and thus remains a major reason of non-relapse mortality. There is a high need for the development of more effective immunomodulatory therapies to prevent and treat GvHD. In this review, we are focusing on advantages and recent challenges of using thymus-derived regulatory T cells (tTreg) to suppress GvHD development.

## GvHD Pathogenesis

In 1966, Billingham initially defined GvHD as a syndrome in which donor immune cells (the allograft) recognize the recipient’s cells and tissues (the host) as foreign, leading to a complex interaction between donor and recipient adaptive immunity followed by massive host tissue destruction (4). The clinical forms of GvHD include acute and chronic GvHD (cGvHD). Acute GvHD (aGvHD) is characterized by a strong systemic inflammation and tissue destruction of multiple organs, particularly the liver, lung, gut, and skin, whereas cGvHD often imitates autoimmune diseases with massive fibrosis of target organs (3, 5). Both GvHD syndromes involve distinct pathological processes. The risk of GvHD development starts during the conditioning phase of the recipient, even before the allograft is infused. Chemotherapy or total-body irradiation of the patient can cause severe tissue damages which activate host antigen-presenting cells (APC). Following antigen presentation, host APC activate CD4^+^ donor T cells in the graft which differentiate into IFN-γ and IL-17 producing T effector cells (6, 7). A strong cytokine response is initiated promoting the recruitment and activation of further effector cells, including NK cells, CD8^+^ T effector cells, and macrophages, leading to organ damages, clinically indicated by a strong aGvHD in the skin, gut, lung, and liver. The occurrence of aGVHD after aHSCT as well as the conditioning regimen itself can furthermore cause tissue destruction of thymic epithelium, resulting in a reduced negative selection of alloreactive CD4^+^ T cells. The release of fibrogenic cytokines such as IL-2, IL-10, and TGF-β activates macrophages which then stimulate the proliferation of tissue fibroblast, leading to massive fibrosis of target organs in cGvHD. In addition, chronic inflammation and the continuous production of inflammatory cytokines such as IL-6 and TNF-α inhibits the generation of induced regulatory T cells (iTreg) as the naïve CD4^+^ T cells preferentially differentiate into T effector cells, and inflammatory cytokines block the suppressive function of tTreg (8–11). Therefore, tTreg should be used as a cellular drug as early as possible in order to achieve the greatest possible therapeutic effect by restoring the immunological balance.

## Treg can Prevent GvHD Development

Up to now, systemic corticosteroid therapy remains the first line treatment for GvHD. The need for new or improved therapies based on manipulating immune responses has extremely increased in the last decades, especially in cases of steroid-refractory GvHD patients. Targeting and modulating T cell responses, the etiological factors in GvHD induction, seems to be a promising strategy.

Thymus-derived Treg, comprising 2–5% of all peripheral blood cells in humans (12), are key players in the modulation of immune responses and play an important role in self-tolerance (13–15). Additionally, tTreg are a mandatory cell type for the maintenance of immune tolerance and thus prevention of overshooting immune responses such as of GvHD after aHSCT (16). The therapeutic efficacy of adoptively transferred tTreg in promoting tolerance has been shown in mouse models of aHSCT (17, 18) and the function of tTreg in reducing the risk of GvHD development has been furthermore demonstrated in humans (8, 19). A high number of tTreg in blood stem cell transplants is associated with reduced risk of GvHD development and patients with active cGvHD show reduced tTreg frequencies compared with healthy volunteers. Therefore, adoptive tTreg transfer to enhance tTreg frequencies in transplanted patients that suppress GvHD development is an attractive therapeutic option and protocols for effective generation of such cellular therapies have been developed.

A significant challenge in the development of efficient tTreg cellular therapies is the low rate of these cells in the peripheral blood. Since in murine models high numbers of tTreg are needed for a marked reduction of GvHD, it has been postulated that ratios of nearly 1:1 tTreg to T effector cells are necessary for successful GvHD prevention (17, 20). However, such high cell numbers of tTreg cannot be isolated from normal blood products including leukapheresis. As one approach, an expansion of isolated tTreg is necessary in order to be able to produce sufficient cell numbers for GvHD prevention in patients.

## *In vitro* Expanded tTreg as Cellular Therapeutic for GvHD Suppression

Human tTreg do not express an exclusive surface marker which allows their isolation without contamination with conventional T cells. However, to achieve a high-quality product for cell expansion, high purity of tTreg is needed as a starting population. The most extensively used method to isolate human tTreg is based on the use of anti-CD25 immunomagnetic beads (21–23). By using this technique, tTreg purities of 50–80% can be achieved. Since CD25 is expressed on tTreg as well as on activated T effector cells, contamination with these cells cannot be completely prevented using anti-CD25 beads and the risk that these T cells will also be activated and expanded after anti-CD3/CD28 antibody stimulation cannot be excluded. The additional use of CD127 as a marker can improve the purity of tTreg (CD4^+^CD25^+^CD127^low^) and their efficacy *in vivo* (24, 25). Nevertheless, expansion results in significant changes of many marker molecules used for tTreg characterization. As an example, a part of the isolated cells loose Foxp3 expression during expansion (26). Furthermore, Voo et al. have shown that human Foxp3^+^ T cell populations also contain Th17 precursors that expand after polyclonal stimulation (27). Therefore, it is difficult to define the real ratio of functionally stable and active tTreg after *ex vivo* expansion. To increase tTreg purity, several groups improved their expansion protocols by adding rapamycin in order to reduce the unwanted proliferation of T effector cells and to increase the stability of expanded tTreg (22, 28).

Despite the difficulties with the functional stability of expanded tTreg and the potential contamination with T effector cells, the adoptive transfer of tTreg for treatment of GvHD is very attractive in order to address the high unmet medical need. Within the last decade, three trials of adoptive tTreg therapy in GvHD patients have been carried out. In 2009, Trzonkowski et al. reported the first-in-man trial which included two GvHD patients (29). The first patient suffered from cGvHD two years after aHSCT and received high doses of corticosteroids [triple-drug therapy; prednisone, tacrolimus, and mycophenolate mofetil (MMF)]. After infusing a single dose of 1 × 10^5^/kg *ex vivo* expanded tTreg, MMF therapy was completely withdrawn, and lung function, blood hemoglobin, and body weight improved. The second patient, however, continued to suffer from aGvHD despite three infusions of 3 × 10^6^/kg expanded tTreg. Finally, the condition further deteriorated and the patient died from multiorgan dysfunction. Di Ianni et al. published clinical data demonstrating the effect of tTreg infusion in the prevention of GvHD in 26 patients with high-risk hematological malignancies (30). In contrast to Trzonkowski et al., Di Ianni used freshly isolated donor tTreg without *ex vivo* expansion. Cellular tTreg infusions started four days prior transplantation with haploidentical CD34^+^ stem cells added with donor T effector cells without any post-transplant immunosuppressive drugs. tTreg to T effector cell ratios of 2:1 were infused with no observable toxicities. Adoptive tTreg transfer was safe and did not diminish the graft versus leukemia effect of co-transferred effector T cells. Only 2 of 26 patients developed an aGvHD and no cGvHD was observed in the first year after treatment. Interestingly, the authors found an improvement in the immunological reconstitution of patients after tTreg transfer, and the relapse rate in this study was only 5% compared with 30–35% seen normally in patients with high-risk leukemia. These impressive results demonstrated that transfer of freshly isolated tTreg is safe and can significantly suppress GvHD development without hampering/affecting the graft versus tumor response.

Compared with peripheral blood, umbilical cord blood contains significant lower amounts of CD4^+^CD25^+^ T effector cells and a greater percentage of CD4^+^CD25^+^CD45RA^+^ tTreg, a subset with a higher stability upon expansion. This makes cord blood an attractive starting product for tTreg expansion (31). Brunstein et al. completed the first clinical trial using two infusions with cord blood expanded tTreg for treatment of 23 patients early after transplantation (32). The partial HLA-matched tTreg were infused on days 1 and 15. All patients underwent additional GvHD prophylaxis with MMF combined with cyclosporine or sirolimus. Patients were treated with doses from 3 to 100 × 10^6^ expanded tTreg/kg with a median purity of Foxp3^+^CD127^−^ cells of 87%. To evaluate the impact of tTreg infusions, the authors compared the risk of graft failure, general mortality, and GvHD development to 108 historical patients with identical transplant regimes but without tTreg therapy. Again, no infusional toxicities were observed but a significant reduction of aGvHD from 61% in historical controls to 43% in patients with tTreg infusions. No enhanced risks in relapse, opportunistic infections or early mortality were observed. These data confirm that adoptive transfer of Treg cellular products as a prophylaxis against GvHD is safe and effective. Nevertheless, one has to keep in mind that the source of cord blood for the large-scale preparation of Treg remains limited.

Theil et al. firstly described the outcome of a tTreg infusion therapy for a limited number of patients with existing cGvHD (33). They infused an average of 2.4 × 10^6^ Treg/kg with an average purity of 84.1% of Foxp3^+^CD127^−^ cells. Treg were infused after a median time of 35 months after transplantation with continued prednisolone treatment. No infusional toxicity or other adverse effects were observed. Two of five patients showed a clinical response with improvement of GvHD symptoms, the other three a stable disease for up to 21 months. These data suggest that cellular Treg therapy may be clinically effective even after years of immunosuppressive therapy and cGvHD.

Nevertheless, functional stability of expanded tTreg and their persistence and distribution following infusions in patients are not sufficiently characterized. The reproducible generation of sufficient quantities of tTreg with high quality and purity currently requires specialized expertise which limits its general applicability to a few specialized transplant centers. Thus, if the immunosuppressive activity of the tTreg product is significantly increased and the complex and uncertain *in vitro* expansion could be avoided, the applicability of the tTreg therapy would be significantly improved.

## Tolerance Induction by Anti-CD4 Stimulation

Like conventional T cells, tTreg require T cell receptor (TCR) stimulation and costimulation for functional activation. Without this stimulation, only the few percent alloreactive tTreg are functionally activated and effective suppressor cells. Reagents that allow a polyclonal activation of tTreg without stimulation of conventional T cells would shift the balance in favor of the tTreg and significantly reduce the necessary number of tTreg needed for GvHD suppression. CD4-mediated activation of tTreg is such a possibility (34).

It is a well-known phenomenon first described by Waldmann’s group that short-time therapy with non-depleting anti-CD4 antibodies can induce long-lasting tolerance (35, 36). They showed that a co-receptor blockade by anti-CD4 antibodies induce the conversion of naïve CD4^+^ T cells into induced Foxp3^+^ Treg. The induced tolerance by such treatment is “dominant, transferable to naïve recipients, and transferred CD4^+^ T cells have the ability to “infect” naïve T cells to acquire a tolerant state” (36). The effect of anti-CD4 antibody treatment on tTreg was not clear at that time. Therefore, we investigated anti-CD4 stimulation on tTreg and found that human CD4^+^CD25^+^Foxp3^+^ tTreg can be functionally activated by anti-CD4 stimulation in a dose-dependent manner (34). CD4-activated tTreg suppress the proliferation and cytokine production of CD4^+^ and CD8^+^ T effector cells. In contrast, anti-CD4 stimulation did not induce suppressive activity in conventional CD4^+^ T cells. The CD4 signal induces a specific phosphorylation of TCR associated signaling molecules (37), sufficient to activate the suppressive function of tTreg but inefficient in activating T effector cells. In contrast to TCR stimulation, the CD4 signal induces no proliferation of tTreg. These findings suggested also a direct activation of tTreg in the course of an anti-CD4 treatment *in vivo*. Additional studies by Kendal et al. firstly demonstrated that tTreg are essential for infectious tolerance induced by non-depleting anti-T cell antibodies (38).

## Gp120 for Therapeutic Activation of Treg Prevents GvHD

**Figure 1:**
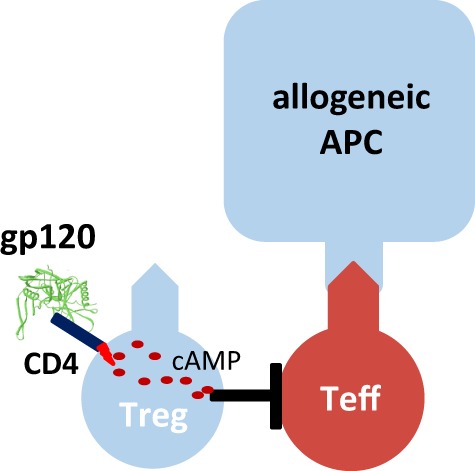
Gp120-mediated activation of human regulatory T cells (Treg). Recombinant gp120 binds to CD4 molecules on the surface of Treg, induces cytosolic cyclic adenosine monophosphate (cAMP) production and thus activates the suppressive function of Treg. Gp120-mediated activation is independent of T cell receptor stimulation. Therefore, gp120 is a polyclonal Treg activator. Inhibition of the tyrosine kinase Lck or blockade of adenylate cyclase activity prevents gp120-mediated cAMP production in Treg and their functional activation.

One molecule that binds with particularly high affinity to human CD4 is the human immunodeficiency virus-1 envelope protein gp120 (Figure 1). We demonstrated that gp120 upon binding to and signaling through CD4 efficiently activates human tTreg (39). Gp120-stimulated tTreg up-regulate cyclic adenosine monophosphate (cAMP), a key event of tTreg activation, and tTreg-mediated suppression (40). Blocking of adenylate cyclases repressed cAMP up-regulation and abrogated suppressor activity in gp120-stimulated tTreg, demonstrating that cAMP up-regulation is crucial for the CD4-mediated suppressive capacity of human tTreg (39).

To investigate the potential tTreg-stimulating properties of gp120 *in vivo*, we used a well-established xenogeneic GvHD model based on the transfer of human peripheral immune cells into immunodeficient mice. Intraperitoneal injection of human PBMC into newborn NOD/*Scid* or Rag2γc^−/−^ mice resulted in development of a lethal GvHD leading to death of mice after 20–90 days, depending on the mouse strain, and the number of transferred PBMC (39, 41). GvHD in mice was characterized by decelerated growth, reduced body weight, and chronic inflammation of skin, liver, and colon thus resembling to symptoms in human GvHD patients. GvHD is induced by the activation and expansion of CD4^+^ T cells which differentiate into IFN-γ and IL-17-forming T effector cells, respectively. The limited number of intrinsic tTreg within the PBMC cannot prevent GvHD development. Transfer of increased ratios of tTreg (PBMC:tTreg 4:1–10:1) blocked the activation, differentiation, and expansion of CD4^+^ T cells and subsequently prevented all signs of GvHD, demonstrating that this human/mouse chimeric animal model is applicable for the analysis of human tTreg function, and tTreg cellular therapeutics *in vivo*. In accordance with published observations, transferred tTreg ratios lower than 10:1 were not effective in GvHD prevention. These results confirm the necessity of high tTreg numbers for successful suppression of GvHD.

Our *in vitro* experiments showed that CD4-mediated stimulation of tTreg by gp120 significantly improves their suppressor function. Consequently, significantly lower tTreg ratios were sufficient to inhibit a mixed leukocyte reaction *in vitro*. Therefore, we postulated that lower tTreg ratios should also be sufficient to prevent GvHD development *in vivo* after CD4-mediated activation of Treg. We investigated this possibility by directly using gp120 as tTreg activator in the humanized GvHD mouse model. Indeed, a single injection of 5 µg gp120 with 5 × 10^6^ PBMC, without additional transfer of tTreg, was able to completely suppress the formation of GvHD (39). Activation of the limited number of intrinsic tTreg within the PBMC was efficient to prevent the activation of pathologic CD4^+^ T cells. Furthermore, gp120 therapy blocked their differentiation into T effector cells, suppressed all signs of GvHD, and induced a long-lasting state of tolerance. However, gp120 therapy is strictly dependent on the presence of tTreg and showed no effect when the small number of intrinsic tTreg were depleted before transfer of PBMC. These results confirmed the *in vitro* data that binding of gp120 to conventional CD4^+^ T cells did not block their activation directly.

The data suggest that gp120-activated tTreg are at least 20 times more effective than resting tTreg. For a cellular tTreg therapy, this means that significantly less tTreg must be transferred in order to achieve the same suppressive effect *in vivo*. Since with today’s protocols more than 10^8^ tTreg can be isolated from a single apheresis, the gp120 stimulation could replace a time- and cost-intensive expansion of the cellular tTreg product.

## Summary

In the last years, cellular tTreg therapy has arrived in clinical testing and could be the first curative treatment form of GvHD in the future. In contrast to current immunosuppressive drugs, often combined with severe adverse side effects due to general immunosuppression, short-time tTreg therapy has the potential to induce long-lasting tolerance without further treatment. The study results published so far are impressive. Significant progress has also been made in the manufacture of cellular tTreg products in recent years. However, the *in vitro* expansion, which is difficult to standardize, is still a significant obstacle to a broad and reproducible clinical application of this cellular product. The CD4-mediated functional activation of tTreg could provide an important contribution to avoid *in vitro* expansion since this activation significantly enhances the suppressive properties of tTreg and therefore reduces the necessary number of cells for efficient GvHD inhibition *in vivo*.

In the meantime, the first closed systems for the production of cell products are available. However, significant improvements still have to be made. The new technologies have to ensure a standardized and reproducible production of the cell products with the same quality and quantity outside cleanroom laboratories in specialized centers, comparable with the production of leukaphereses in transfusion centers. This attractive and promising therapy can only be offered to all transplant patients, when the cell therapeutic itself can be produced in the same quality, quantity, and functionality at different locations.

## Author Contributions

HJ and JS contributed to the conception, design, writing, and revision of the manuscript. CB and AT contributed to the writing and revision of the manuscript.

## Conflict of Interest Statement

The authors declare that the research was conducted in the absence of any commercial or financial relationships that could be construed as a potential conflict of interest. However, HJ is a co-inventor on patents [EP2109770 (A2) and EP2332991 (A3)] that are in connection with the use of CD4-dependent activation of human tTreg and their potential clinical use.

## References

[1] Blazar BR Murphy WJ Abedi M. Advances in graft-versus-host disease biology and therapy. Nat Rev Immunol (2012) 12(6):443–58. 10.1038/nri3212 22576252 PMC3552454

[2] Gratwohl A Pasquini MC Aljurf M Atsuta Y Baldomero H Foeken L One million haemopoietic stem-cell transplants: a retrospective observational study. Lancet Haematol (2015) 2(3):E91–100. 10.1016/S2352-3026(15)00028-9 26687803

[3] Wolff D Gerbitz A Ayuk F Kiani A Hildebrandt GC Vogelsang GB Consensus Conference on clinical practice in chronic graft-versus-host disease (GVHD): first-line and topical treatment of chronic GVHD. Biol Blood Marrow Tr (2010) 16(12):1611–28. 10.1016/j.bbmt.2010.06.015 20601036

[4] Billingham RE The biology of graft-versus-host reactions. Harvey Lect (1966) 62:21–78. 4875305

[5] Dhir S Slatter M Skinner R. Recent advances in the management of graft-versus-host disease. Arch Dis Child (2014) 99(12):1150–7. 10.1136/archdischild-2013-304832 25016613

[6] Sakoda Y Hashimoto D Asakura S Takeuchi K Harada M Tanimoto M Donor-derived thymic-dependent T cells cause chronic graft-versus-host disease. Blood (2007) 109(4):1756–64. 10.1182/blood-2006-08-042853 17032915

[7] Coghill JM Sarantopoulos S Moran TP Murphy WJ Blazar BR Serody JS Effector CD4(+) T cells, the cytokines they generate, and GVHD: something old and something new. Blood (2011) 117(12):3268–76. 10.1182/blood-2010-12-290403 21245483 PMC3069668

[8] Zorn E Kim HT Lee SJ Floyd BH Litsa D Arumugarajah S Reduced frequency of FOXP3(+) CD4(+)CD25(+) regulatory T cells in patients with chronic graft-versus-host disease. Blood (2005) 106(8):2903–11. 10.1182/blood-2005-03-1257 15972448 PMC1895303

[9] Rieger K Loddenkemper C Maul J Fietz T Wolff D Terpe H Mucosal FOXP3(+) regulatory T cells are numerically deficient in acute and chronic GvHD. Blood (2006) 107(4):1717–23. 10.1182/blood-2005-06-2529 16278306

[10] Chen X Das R Komorowski R Beres A Hessner MJ Mihara M Blockade of interleukin-6 signaling augments regulatory T-cell reconstitution and attenuates the severity of graft-versus-host disease. Blood (2009) 114(4):891–900. 10.1182/blood-2009-01-197178 19491393 PMC2716024

[11] Das R Chen X Komorowski R Hessner MJ Drobyski WR. Interleukin-23 secretion by donor antigen-presenting cells is critical for organ-specific pathology in graft-versus-host disease. Blood (2009) 113(10):2352–62. 10.1182/blood-2008-08-175448 19059877 PMC2652376

[12] Jonuleit H Schmitt E Stassen M Tuettenberg A Knop J Enk A. Identification and functional characterization of human CD4(+)CD25(+) T cells with regulatory properties isolated from peripheral blood. J Exp Med (2001) 193(11):1285–94. 10.1084/jem.193.11.1285 11390435 PMC2193380

[13] Sakaguchi S Regulatory T cells: key controllers of immunologic self-tolerance. Cell (2000) 101(5):455–8. 10.1016/S0092-8674(00)80856-9 10850488

[14] Shevach EM McHugh RS Piccirillo CA Thornton AM Control of T-cell activation by CD4(+) CD25(+) suppressor T cells. Immunol Rev (2001) 182:58–67. 10.1034/j.1600-065X.2001.1820104.x 11722623

[15] Jonuleit H Adema G Schmitt E. Immune regulation by regulatory T cells: implications for transplantation. Transpl Immunol (2003) 11(3–4):267–76. 10.1016/S0966-3274(03)00057-1 12967780

[16] Beres AJ Drobyski WR The role of regulatory T cells in the biology of graft versus host disease. Front Immunol (2013) 4:163 10.3389/fimmu.2013.00163 23805140 PMC3690651

[17] Taylor PA Lees CJ Blazar BR. The infusion of ex vivo activated and expanded CD4(+)CD25(+) immune regulatory cells inhibits graft-versus-host disease lethality. Blood (2002) 99(10):3493–9. 10.1182/blood.V99.10.3493 11986199

[18] Edinger M Hoffmann P Ermann J Drago K Fathman CG Strober S CD4(+)CD25(+) regulatory T cells preserve graft-versus-tumor activity while inhibiting graft-versus-host disease after bone marrow transplantation. Nat Med (2003) 9(9):1144–50. 10.1038/nm915 12925844

[19] Rezvani K Mielke S Ahmadzadeh M Kilical Y Savani BN Zeilah J High donor FOXP3-positive regulatory T-cell (T-reg) content is associated with a low risk of GVHD following HLA-matched allogeneic SCT. Blood (2006) 108(4):1291–7. 10.1182/blood-2006-02-003996 16627754 PMC1895877

[20] Taylor PA Panoskaltsis-Mortari A Swedin JM Lucas PJ Gress RE Levine BL L-Selectin(hi) but not the L-selectin(lo) CD4(+)25(+) T-regulatory cells are potent inhibitors of GVHD and BM graft rejection. Blood (2004) 104(12):3804–12. 10.1182/blood-2004-05-1850 15292057

[21] Brunstein CG Miller JS McKenna DH Hippen KL Defor TE Sumstad D Umbilical cord blood-derived T regulatory cells to prevent GVHD: kinetics, toxicity profile, and clinical effect. Blood (2016) 127(8):1044–51. 10.1182/blood-2015-06-653667 26563133 PMC4768428

[22] Mckenna DH Sumstad D Kadidlo DM Batdorf B Lord CJ Merkel SC Optimization of cGMP purification and expansion of umbilical cord blood-derived T-regulatory cells in support of first-in-human clinical trials. Cytotherapy (2017) 19(2):250–62. 10.1016/j.jcyt.2016.10.011 27887864 PMC5237605

[23] Velaga S Alter C Dringenberg U Thiesler CT Kuhs S Olek S Clinical-grade regulatory T cells: comparative analysis of large-scale expansion conditions. Exp Hematol (2017) 45:27–35. 10.1016/j.exphem.2016.09.008 27693388

[24] Nadig SN Wieckiewicz J Wu DC Warnecke G Zhang W Luo SQ In vivo prevention of transplant arteriosclerosis by ex vivo-expanded human regulatory T cells. Nat Med (2010) 16(7):809–13. 10.1038/nm.2154 20473306 PMC2929438

[25] Di Ianni M Del Papa B Zei T Ostini RI Cecchini D Cantelmi MG T regulatory cell separation for clinical application. Transfus Apher Sci (2012) 47(2):213–6. 10.1016/j.transci.2012.06.007 22795999

[26] Hoffmann P Boeld TJ Eder R Huehn J Floess S Wieczorek G Loss of FOXP3 expression in natural human CD4(+)CD25(+) regulatory T cells upon repetitive in vitro stimulation. Eur J Immunol (2009) 39(4):1088–97. 10.1002/eji.200838904 19283780

[27] Voo KS Wang YH Santori FR Boggiano C Wang YH Arima K Identification of IL-17-producing FOXP3(+) regulatory T cells in humans. Proc Natl Acad Sci USA (2009) 106(12):4793–8. 10.1073/pnas.0900408106 19273860 PMC2653560

[28] Hippen KL Riley JL June CH Blazar BR. Clinical perspectives for regulatory T cells in transplantation tolerance. Semin Immunol (2011) 23(6):462–8. 10.1016/j.smim.2011.07.008 21820917 PMC3230779

[29] Trzonkowski P Bieniaszewska M Juscinska J Dobyszuk A Krzystyniak A Marek N First-in-man clinical results of the treatment of patients with graft versus host disease with human ex vivo expanded CD4+CD25+CD127-T regulatory cells. Clin Immunol (2009) 133(1):22–6. 10.1016/j.clim.2009.06.001 19559653

[30] Di Ianni M Falzetti F Carotti A Terenzi A Castellino F Bonifacio E Tregs prevent GVHD and promote immune reconstitution in HLA-haploidentical transplantation. Blood (2011) 117(14):3921–8. 10.1182/blood-2010-10-311894 21292771

[31] Godfrey WR Spoden DJ Ge YG Baker SR Liu B Levine BL Cord blood CD4(+)CD25(+)-derived T regulatory cell lines express FoxP3 protein and manifest potent suppressor function. Blood (2005) 105(2):750–8. 10.1182/blood-2004-06-2467 15374887

[32] Brunstein CG Miller JS Cao Q McKenna DH Hippen KL Curtsinger J Infusion of ex vivo expanded T regulatory cells in adults transplanted with umbilical cord blood: safety profile and detection kinetics. Blood (2011) 117(3):1061–70. 10.1182/blood-2010-07-293795 20952687 PMC3035067

[33] Theil A Tuve S Oelschlagel U Maiwald A Dohler D Ossmann D Adoptive transfer of allogeneic regulatory T cells into patients with chronic graft-versus-host disease. Cytotherapy (2015) 17(4):473–86. 10.1016/j.jcyt.2014.11.005 25573333

[34] Becker C Kubach J Wijdenes J Knop J Jonuleit H CD4-mediated functional activation of human CD4(+)CD25(+) regulatory T cells. Eur J Immunol (2007) 37(5):1217–23. 10.1002/eji.200636480 17407195

[35] Qin SX Cobbold SP Pope H Elliott J Kioussis D Davies J Infectious transplantation tolerance. Science (1993) 259(5097):974–7. 10.1126/science.8094901 8094901

[36] Waldmann H Adams E Cobbold S. Reprogramming the immune system: co-receptor blockade as a paradigm for harnessing tolerance mechanisms. Immunol Rev (2008) 223:361–70. 10.1111/j.1600-065X.2008.00632.x 18613847

[37] Helling B Koenig M Daelken B Engling A Kroemer W Heim K A specific CD4 epitope bound by tregalizumab mediates activation of regulatory T cells by a unique signaling pathway. Immunol Cell Biol (2015) 93(4):396–405. 10.1038/icb.2014.102 25512343 PMC4407014

[38] Kendal AR Chen Y Regateiro FS Ma JB Adams E Cobbold SP Sustained suppression by Foxp3(+) regulatory T cells is vital for infectious transplantation tolerance. J Exp Med (2011) 208(10):2043–53. 10.1084/jem.20110767 21875958 PMC3182049

[39] Becker C Taube C Bopp T Becker C Michel K Kubach J Protection from graft-versus-host disease by HIV-1 envelope protein gp120-mediated activation of human CD4(+)CD25(+) regulatory T cells. Blood (2009) 114(6):1263–9. 10.1182/blood-2009-02-206730 19439734

[40] Bopp T Becker C Klein M Klein-Hessling S Palmetshofer A Serfling E Cyclic adenosine monophosphate is a key component of regulatory T cell mediated suppression. J Exp Med (2007) 204(6):1303–10. 10.1084/jem.20062129 17502663 PMC2118605

[41] Hahn SA Stahl HF Becker C Correll A Schneider FJ Tuettenberg A Soluble GARP has potent antiinflammatory and immunomodulatory impact on human CD4(+) T cells. Blood (2013) 122(7):1182–91. 10.1182/blood-2012-12-474478 23818544

